# Nonlinear light absorption in Ti_3_C_2_T_*x*_ MXene: a theoretical study

**DOI:** 10.1039/d5ra01751c

**Published:** 2025-07-10

**Authors:** Hayk Minassian, Armen Melikyan, Manuel Rodrigues Gonçalves, Petros Petrosyan

**Affiliations:** a A. Alikhanian National Science Laboratory Alikhanyan Str. Build. 2 0036 Yerevan Armenia hminassian@yerphi.am; b Institute of Applied Problems of Physics of NAS 25, Hr. Nersessian Str. 0014 Yerevan Armenia armen_melikyan@iapp.am; c Ulm University – Institute of Experimental Physics Albert-Einstein-Allee 11 89081 Ulm Germany manuel.goncalves@uni-ulm.de; d Yerevan State University 1 Alek Manukyan Str. 0025 Yerevan Armenia petrosyanpetros00@gmail.com

## Abstract

Nonlinear absorption of MXenes has been investigated experimentally in several recent publications, and applications of these new 2D materials in broadband ultrafast photonics has already been demonstrated. In particular, MXenes have been used in the generation of highly stable femtosecond pulses in mode-locked fiber lasers. The optical nonlinearities appearing in different MXenes in the vis-NIR wavelength range have been explained theoretically based on the theory of saturation of nonlinear absorption in a two-level atomic system. This approach requires a fitting procedure applied to determine the saturation intensity of the MXene. However, this approximation does not account for the band structure of MXene, and therefore the microscopic character of the saturation of nonlinear absorption is still not understood. In this communication we use published results of calculations of the band structure of titanium carbide MXene and apply the density matrix theory to calculate the nonlinear absorption coefficient for a two-band model, in the resonance approximation. The latest experimental results on nonlinear transmittance at pump wavelengths between 1000 nm and 1500 nm are discussed in this framework and the saturation dynamics of absorption is revealed.

## Abstract 2

We use published results of calculations of the band structure of titanium carbide MXene and apply the density matrix theory to calculate the nonlinear absorption coefficient for a two-band model, in the resonance approximation.

## Graphical abstract

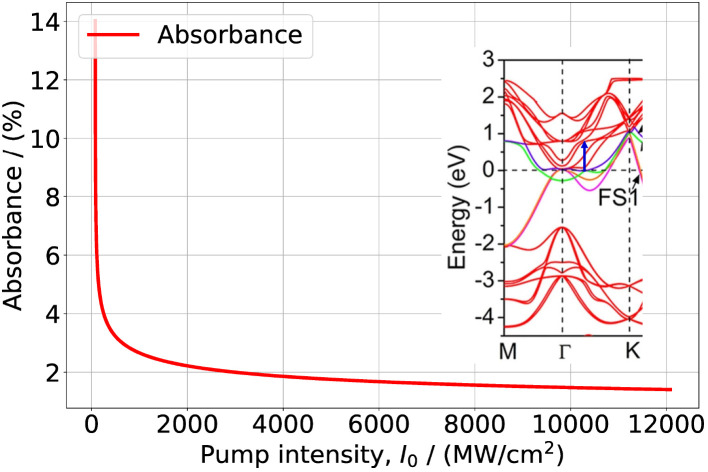

## 1. Introduction

Around a decade ago, a new family of 2D materials was discovered – carbides and nitrides of transition metals, called MXenes.^1,2^ The investigation of the linear optical properties of MXenes has been carried out by electron energy loss spectroscopy (EELS) and optical measurements in the visible, UV, and IR ranges.^3–6^ The analysis of experimental data allowed identifying bulk plasmons, surface plasmons (SPs) as well as interband transitions (IBT) in these 2D metal carbides and nitrides.^7^ Due to their unique optoelectronic properties as well as metal-like electronic conductivity, MXenes became the subject of intensive research.^8,9^ Theoretical studies of the linear optical properties were realized in ref. 10–12, where IBT, quadrupole surface plasmons (QSP) and transversal dipole surface plasmons (TDSP) were identified as the main mechanisms of linear absorption in the vis-NIR wavelength range. Although, in the spectral range of *λ* = 600–1600 nm different resonances arise, the absorption curve of Ti_3_C_2_T_*x*_ does not present sharp peaks. This behavior is interpreted by partial overlapping of these resonances,^11,13^ as well as due to noticeable broadening caused by rather short electron–electron relaxation times.^14–16^

Nonlinear optical absorption properties of MXenes have been discovered more recently. The first experimental studies on optical nonlinearities in 2D titanium carbide and carbonitride MXenes (Ti_3_C_2_T_*x*_ and Ti_3_CNT_*x*_, where T_*x*_ represents functional groups such as –OH and –F) suggest that MXenes exhibit robust nonlinear optical properties.^17–19^ Some of them are already being utilized in fiber-based femtosecond lasers for mode locking.^20^

Identified by numerous experimental groups,^17–19,21–39^ the optical nonlinearities in different MXenes in the vis-NIR wavelength range are interpreted in terms of saturation of nonlinear absorption (NLA) in a two-level system (TLS). In these studies, the saturation intensity is found by fitting the experimental data by the well-known expression for the NLA coefficient in a TLS model. However, the mechanisms for the manifestation of nonlinearity in the TLS model and in the case of a solid, are different. Therefore, a theoretical study of the nonlinear optical properties of MXenes requires accounting for the band structure of the charge carriers. In this communication we calculate the NLA coefficient of the most studied MXene, Ti_3_C_2_T_*x*_, based on the density matrix formalism. The theory developed here allows the interpretation of experimental data on NLA in Ti_3_C_2_T_*x*_, in the wavelength range *λ* = 600–1600 nm, without introducing fitting parameters.

### 1.1. Features of the Ti_3_C_2_T_*x*_ band structure responsible for the nonlinear optical processes

To interpret the NLA experimental data of MXene, using the two-level atomic model, a fitting procedure is necessary. Several fitting parameters have been used based on experimental investigations, namely the saturation intensity of the nonlinear absorption, the modulation depth, and the nonsaturable loss. The theory presented below allows the calculation of the NLA coefficient, using only experimental data of a complex dielectric function, the linear absorption coefficient and the charge carrier relaxation times in the MXene.

The first theoretical studies of the band structure of Ti_3_C_2_T_*x*_ based on density functional theory (DFT) were obtained for free standing monolayers and either metallic-like, or semiconductor-like behavior was revealed.^40,41^ In particular, it was shown that Ti_3_C_2_F_2_ and Ti_3_C_2_(OH)_2_ can be either metals, or narrow-band gap semiconductors, depending strongly on how the surface groups F and OH are geometrically terminated. Although most of the DFT calculations of band structures of MXenes were done for monolayers, there are also DFT calculations for multilayer (stacked) MXene structures.^31,42,43^ In these articles, the main features of the band structure of the stacked Ti_3_C_2_(OH)_2_ sheets, in the wavelength range 1000 nm to 1500 nm, are similar. In this article we use the data from ref. 42, where the band structure for a bilayer and multilayers was calculated. Moreover, calculations of the band structure of a stacked sample and a bilayer of Ti_3_C_2_(OH)_2_ were done, based on dispersion-corrected density functional theory (DFT-D), in which long-range dispersion interactions are taken into consideration. It was realized that at the Fermi energy, *E*_F_, the density of states (DOS) mainly originates from the nearly free 3d electron states of Ti. Correspondingly, the 3d electrons of Ti contribute predominantly to the electronic conduction since the electronic transport properties are governed by the electrons near *E*_F_. It was demonstrated that the intralayer bonding is strong, while the interlayer is weak, since there are electron density dilution zones between neighboring layers. Weak interaction between MXene layers results in small differences in band structures of samples of different thicknesses (*e.g.*, see the band structure of bilayer and stacked Ti_3_C_2_(OH)_2_ in ref. 42).

Since nonlinear optical phenomena manifest themselves most clearly in resonance conditions, it is possible to restrict the model to consider only two bands (two-band model). As the DFT calculations show, in the vis-NIR range there are suitable pairs of bands below and above the Fermi-level, that can provide resonance transitions.^42^

**Fig. 1:**
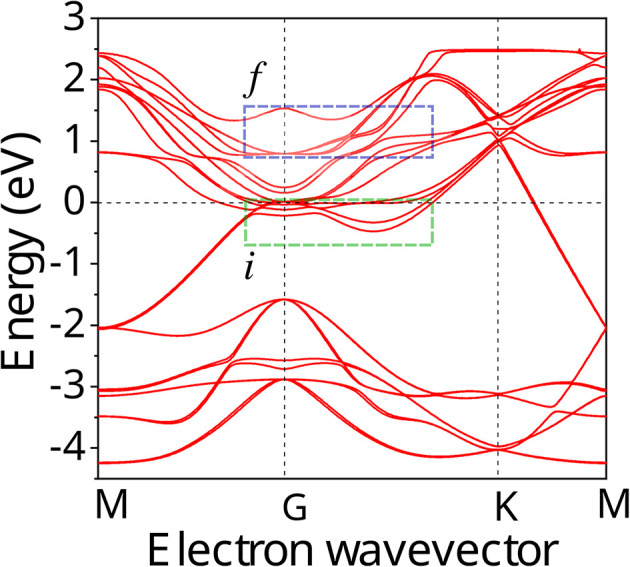
Electronic band structure of the Ti_3_C_2_(OH)_2_ bilayer for the simple hexagonal stacking type of layers. Initial, i and final f, bands are marked, respectively, with green and blue dotted rectangles. Reproduced and adapted from data provided by the authors of ref. 42. Copyright (2015) Springer Nature Publishing Group.

For specific wavelengths of pump laser pulses, the resonant coupled bands in Ti_3_C_2_T_*x*_ MXene are identified from band structure calculations. For example, in Fig. 1 the band structure of a bilayer Ti_3_C_2_(OH)_2_ MXene obtained from DFT calculations is presented.^42^ The accuracy of the results of our calculations presented below is limited by the width of curves of Fig. 1, showing the dispersion structure *E*(*k*).

All the resonance transitions in the wavelength range *λ* = 600–1600 nm in the *k*-space take place in the narrow energy range around the *Γ* point and in the middle of the path *Γ* → *K* of the Brillouin zone. Initial (i, below the Fermi level) and final (f, above the Fermi level) bands that can be resonantly coupled are marked with green and blue dotted rectangles, respectively (see Fig. 1). We note that at points of high-symmetry in Ti_3_C_2_(OH)_2_ the dispersion laws of carriers around *E*_F_ in the wavelength range specified above, can be approximated by parabolic curves of different curvature, corresponding to different effective masses of charge carriers.

Noticeable nonlinear absorption of light in the MXene at a given pump frequency is possible if there is at least one pair of i and f bands between the extremes, of which a direct resonant transition is possible. In addition, a flat (low curvature) parabolas describing dispersion laws corresponds to a high DOS of carriers and provides strong absorption of light. Therefore, it is not surprising that in experiments on NLA in MXenes, the saturation behavior strongly depends on the pump laser wavelength. For example, in ref. 33 it was found that for the wavelengths between *λ* = 1000 nm and *λ* = 1500 nm, using excitation of short laser pulses, the absorption saturation occurs at very low intensities, *I* = 1.29 MW cm^−2^ and *I* = 1.02 MW cm^−2^, respectively. Contrary to this, in ref. 27, the nonlinear transmission of Ti_3_C_2_T_*x*_ MXene measured at *λ* = 1550 nm and *λ* = 2000 nm resulted in highly different saturation intensities of *I* = 10.68 MW cm^−2^ and *I* = 651.23 MW cm^−2^. It is reasonable to assume that such large differences in saturation intensity may be associated with the peculiarities of the MXene band structure. Below, we elaborate on this approach to interpret recent experiments with laser pulses of appropriate pulse width.

Typically, samples with few layers or a stacked multilayer of MXenes offering similar band structures^31,42^ are studied in nonlinear optical experiments. For both structures, in order to calculate the NLA coefficient, it is necessary to determine the effective mass of charge carriers, which can be done using the results of band structure calculations. To do this, each of the selected bands of both samples has to be approximated by a parabolic curve in the vicinity of the symmetry point, allowing to calculate the effective mass of carriers. Below, we apply this method to determine the nonlinear absorption coefficient to a stacked sample of a few layers of Ti_3_C_2_(OH)_2_, since in most cases the termination T_*x*_ of synthesized Ti_3_C_2_T_*x*_ samples is the OH functional group.^44,45^ Moreover, the DFT calculations of the band structures in 42 are performed for Ti_3_C_2_(OH)_2_.

### 1.2. Recent experimental results of nonlinear absorption

Since the theoretical method used in this article is applicable in the steady-state regime, we briefly summarise the application of our method to results of experiments on nonlinear absorption in MXene with long pump laser pulses.

In very recent articles the nonlinear optical properties of samples of Ti_3_C_2_T_*x*_ embedded in PVA were investigated, for films of thickness around *z*_0_ = 70–80 nm,^38^ and for *z*_0_ = 14.1 µm.^39^ In these experiments the nonlinear absorption was measured for *λ* = 1090 nm and *λ* = 1064 nm, respectively. A very large difference in the saturation intensity was found. *I*_sat_ = 1300 MW cm^−2^ in the first case and *I*_sat_ = 2.08 MW cm^−2^ in the second. These values were found by fitting the measured data (see eqn (8) below).

Another interesting case was studied in ref. 37 where the transmittance depending on input intensity for a few layer Ti_3_C_2_T_*x*_ was measured for the laser pulse central wavelength *λ* = 1565 nm. The corresponding fitting of eqn (8) using the measured data, results in the following saturation intensity value *I*_sat_ = 105.28 MW cm^−2^. In ref. 35 for laser pulses with near central wavelength, *λ* = 1550 nm, for the case of a stacked (multilayer) sample, a highly different value of the saturation intensity, *I*_sat_ = 1.5 MW cm^−2^ was obtained. Thus, surprisingly, close to *λ* ∼ 1000 nm, a change in the pump wavelength of only 26 nm, used in thick Ti_3_C_2_T_*x*_ samples, leads to a change in the saturation intensity by three orders of magnitude. The situation is quite different for the pump wavelength around *λ* ∼ 1500 nm, when for two very close wavelengths the saturation regime strongly differs (by two orders of magnitude) for thin (a few layers) and thick (multilayer) samples.

The stronger optical absorption exhibited experimentally and the higher imaginary component of the dielectric constant for thicker Ti_3_C_2_T_*x*_ films, were presented as the reasons for the low values of *I*_sat_.^19^ However, accounting only for this size effects provides just a qualitative analysis of the character of saturation. To obtain a complete picture of absorption, a microscopic approach based on the band structure of MXene with calculated NLA coefficient (see eqn (4) below) must be involved and will be presented in Section 3 below.

## 2. Theoretical model of the nonlinear absorption

### 2.1. Nonlinear absorption coefficient of Ti_3_C_2_(OH)_2_

The calculation of the NLA is based on the density matrix method developed in ref. 46 for a two-band semiconductor excited by the electric field **E**(*z*, *t*) of laser light**E**(*z*, *t*) = **E**_0_(*z*) exp[*i*(*qz* − *ωt*)] + c.c.

The optical wave propagation direction is along the *z*-axis and its amplitude in the medium is (**E**_0_(*z*)), where *q* is the *z*-component of the wave vector of light. For each pump frequency *ω*, we select a pair of resonantly coupled bands: the occupied band, i and the free band, f. Obviously, for a correct description of the nonlinear absorption processes, it is also necessary to account for various processes of electron scattering (interband and intraband), which are non-resonant in nature. These are usually involved in consideration by introducing appropriate relaxation times. Furthermore, accounting only for direct resonance interband transitions (*q* < 10^4^ cm^−^^1^) and neglecting non-resonance interactions, that play a negligible role in nonlinear processes, we obtain the density matrix equations (DME), following the method of Ogasawara.^46^1
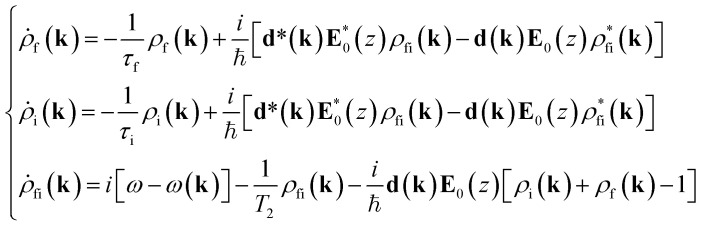
with
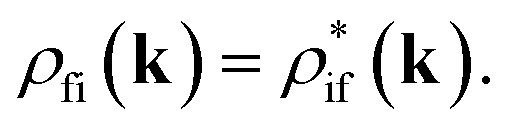


In eqn (1), *ρ*_i_(**k**) and *ρ*_f_(**k**) denote respectively the electron occupation probabilities in the I and f bands, **k** is the wave vector of charge carriers, ℏ*ω*(**k**) = *ε*_0_ + *ε*_f_(**k**) − *ε*_i_(**k**), where *ε*_0_ is the energy separation between resonantly coupled i and f bands at the point *k*_0_ corresponding to the extreme of carrier energies, *ε*_i_(**k**) and *ε*_f_(**k**) are the dispersion laws in the i and f bands. The off-diagonal matrix element *ρ*_fi_(**k**) determines the material polarization, **d**(**k**) is the induced dipole moment directed along **E**_0_(*z*) and, without any loss of generality, we will assume **d**(**k**) = d*(**k**). *T*_2_ is the dipole relaxation time. The parameters *τ*_i_ and *τ*_f_ are the intraband relaxation times,2
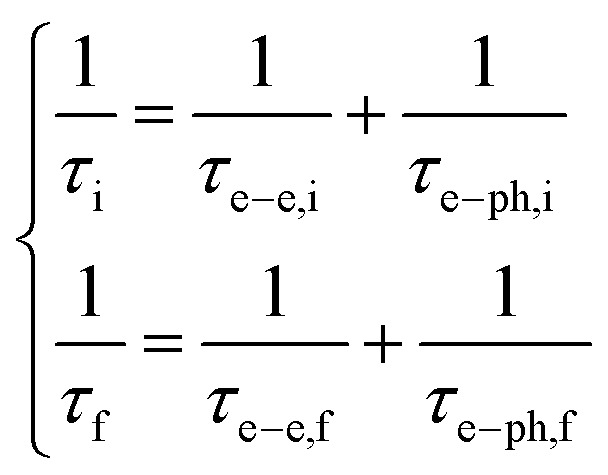
where *τ*_e–e,i_, and *τ*_e–e,f_, are carrier–carrier scattering times, *τ*_e–ph,i_ and *τ*_e–ph,f_ are carrier–phonon scattering times in coupled bands. In eqn (1) the term responsible for recombination of charge carriers is absent, since the relaxation time of the spontaneous emission process *τ*_s_ in Ti_3_C_2_T_*x*_ is much longer than *T*_2_, *τ*_i_, and *τ*_f_.^14–16^ The steady state solution of eqn (1) was used in ref. 47 to describe the saturation of nonlinear absorption in a bulk sample of InGaAsP. We also introduce the intensity of light wave in the absorbing medium *I*(*z*) = (*c*/8π)|*E*_0_(*z*)|^2^, where *c* is the speed of light in vacuum, and *z* is the path coordinate of light in the medium.

To calculate the NLA coefficient *α*[*I*(*z*)] in MXene, we use the well-known definition^47^
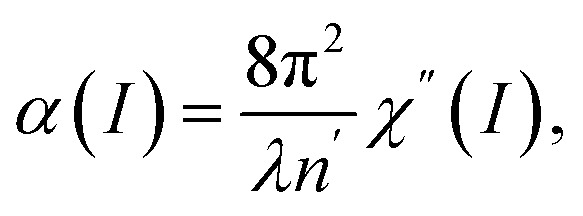
where *λ* is the wavelength of light, *n*′ is the real part of the refractive index, *χ*″ is the imaginary part of susceptibility, defined as follows3
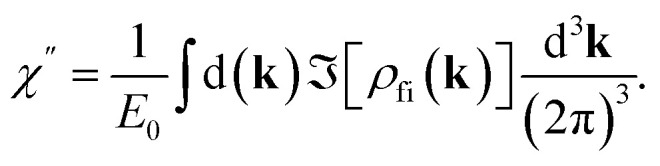


The imaginary part of *ρ*_fi_(**k**) is determined by solving the system of eqn (1). For long enough laser pulses of duration Δ*τ*_pulse_ , we use the steady state solution of eqn (1), obtaining4
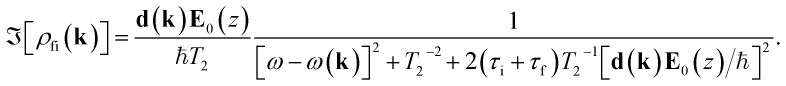


From eqn (4) we can see that the dependence of intensity on *ρ*_fi_(**k**) and consequently also of *α*(*I*) are governed by the dispersion relation of the charge carriers, *ω*(**k**). In further calculations we assume that electron–electron and electron–phonon relaxation times in interacting bands are the same, *τ*_e–e,i_ = *τ*_e–e,f_ and *τ*_e–ph,i_ = *τ*_e–ph,f_.

The calculation of the NLA coefficient *α*(*I*) is obtained from eqn (3) and (4). It is clear that the main contribution to the susceptibility of eqn (3) comes from the narrow region of the energy spectrum in the *k*-space around the wave number *k*_0_, determined from the resonance condition *ω* = *ω*(*k*_0_). Consequently, as usually is accepted in resonance conditions,^46–48^ we ignore the dependence of the transition dipole moment on the electron wavevector, replacing **d**(**k**) by *d*_0_. The quantity *d*_0_ is determined below using the experimental value of the linear absorption coefficient of Ti_3_C_2_T_*x*_.

We must mention that in the range *λ* = 600–1600 nm the resonant interband transitions of Ti_3_C_2_(OH)_2_ (as can be seen in Fig. 1), can take place between the energy bands around the *Γ* point and in the middle of the path *Γ* → *K* of the Brillouin zone. Moreover, these bands are well approximated by parabolic curves. Under these assumptions we obtain from eqn (3) and (4) the following expression for the NLA coefficient, calculated in the two-band model (TBM).5
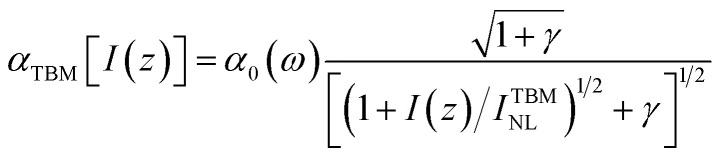
Here the values of *I*(*z*) depending on input intensity *I*_0_, will be determined in the next section by solving the propagation equation containing *α*_TBM_[*I*(*z*)]. In eqn (5)*α*_0_(*ω*) is the linear absorption coefficient of MXene and it takes the form6
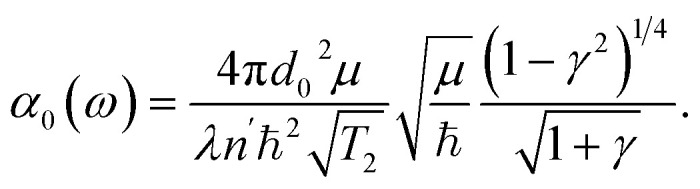
*I*^TBM^_NL_ is a measure of nonlinearity and determined by material parameters of the MXene flake and excitation frequency7
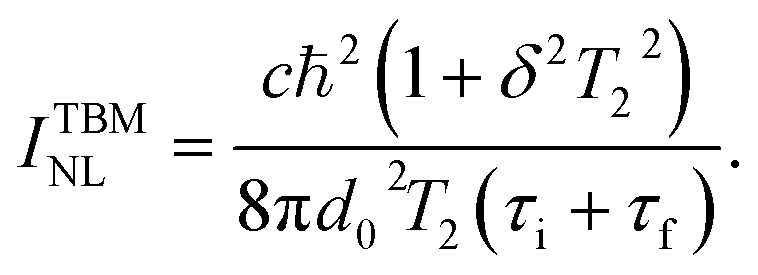


In eqn (6), *µ* = *m*_f_*m*_i_/(*m*_i_ + *m*_f_) is the reduced mass, where *m*_i_ and *m*_f_ are the effective mass determined from the band structure of MXene. The quantity *d*_0_ is calculated from the eqn (6) for the linear absorption coefficient, using measured values of the parameters *α*_0_(*ω*),^49^*T*_2_, *τ*_i_ and *τ*_f_.^14–16^ Moreover, in eqn (5) and (6)
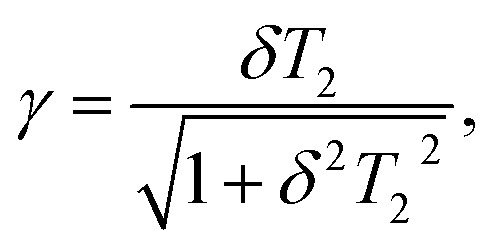
with
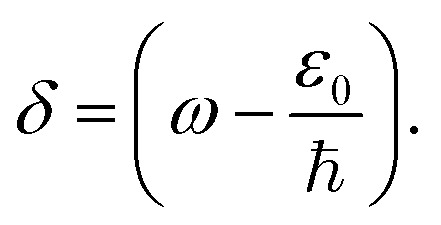


We note that the quantity *δ* can take both positive and negative values, which is a result of the specific band structures of the charge carriers in MXenes. Indeed, as is seen from Fig. 1, alongside resonance transitions between i and f bands having correspondingly negative *ω* > *ε*_0_/ℏ and positive dispersion *ω* < *ε*_0_/ℏ, there are also other i and f bands resonantly coupled, with dispersions of opposite signs. In addition, there are also flat band segments supporting resonance transitions as well. For different experiments with given pump photon energies, this peculiarity is considered in our calculations. Namely, we account for all resonance interband transitions in *k*-space around the *Γ* point and in the middle of the path *Γ* → *K* of the Brillouin zone.

As is seen from eqn (5), the NLA coefficient differs from the one based on the TLS model, widely used to interpret nonlinear optical phenomena in MXenes8
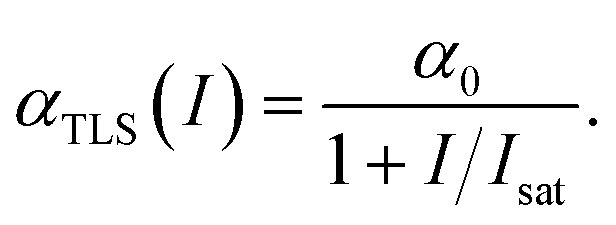


The quantity *I*_sat_ in this expression, called saturation intensity, is considered as an unknown parameter in experimental studies and is revealed by fitting eqn (8) with measured data. We note that the expression of eqn (8) can be obtained using eqn (4) for the particular case of *ω*(**k**) = const. and assigning all relaxation times in eqn (4) to the two-level atom.

### 2.2. Dependence of the transmittance of Ti_3_C_2_T_*x*_ on the excitation intensity

To interpret experimental data on the NLA, we solve the nonlinear propagation equation for the light intensity with the above calculated NLA coefficient of eqn (5),9
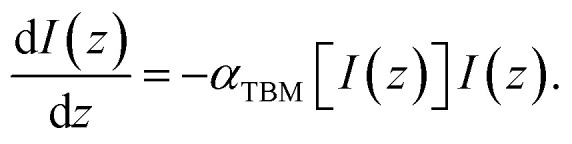


Instead of looking for the function *I*(*z*), we obtain from eqn (9) the inverse function *z*(*I*) that allows to represent the thickness of the sample as a function of the output intensity.10
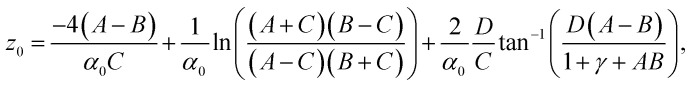
where
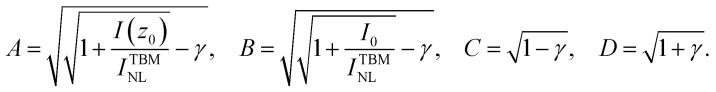


Thus, eqn (9) is solved with respect to thickness of the sample *z*_0_, as a function of the input and output intensities. Furthermore, we introduce the light transmittance of a MXene sample, which is the quantity measured in most of the nonlinear experiments11
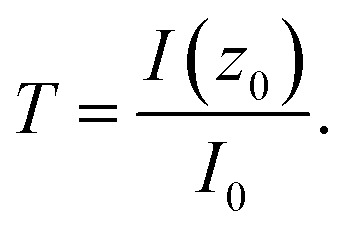


The expression in eqn (11) implicitly represents the output intensity dependence on thickness *z*_0_ of the sample and will be applied in the next sections for the interpretation of NLA experiments. Our simulations, based on eqn (10) for different input intensities, show that an increase in the thickness of the sample leads to a decrease of saturation intensity, in accordance with experiment (see *e.g.* ref. 19). For instance, when the thickness of the MXene sample is increased from 20 nm to 200 nm for *I*_0_ = 10 MW cm^−2^ the absorbance *A* = 1 − *T* changes from 0.03 to 0.32.

We note that for a given wavelength the band structure of MXene may contain resonant transitions between different pairs of bands. In such conditions, the nonlinear absorption coefficient can generally be represented as a sum of the absorption coefficients, *i.e.*12
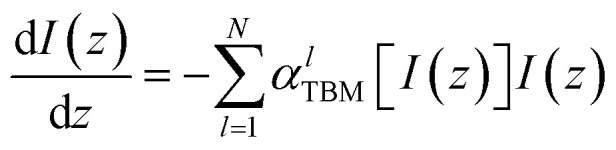
Here *N* indicates the number of resonance transitions at a fixed pump wavelength.

Thus, this approach based on the band structure of MXene allows for a realistic description of the nonlinear optical properties. In contrast to the TLS model, the *I*^TBM^_NL_ in the two-band model is obtained from the solution of DME without introducing any fitting procedure.

## 3. Application of the model to experimental data

### 3.1. Analysis of the experimental NLA data at pump wavelengths 1064 nm and 1090 nm

First, we consider the experiments in ref. 38 and ref. 39, where thick samples were studied when excited at wavelength *λ* = 1090 nm and *λ* = 1064 nm, corresponding to energies *E* = 1.138 eV and *E* = 1.165 eV, respectively. For further analysis we use calculations in the frame of DFT dispersion laws of carriers of thick samples.^42^ First of all, it is seen in Fig. 3(b) of ref. 42 that there are resonance transitions (with different DOS) from bands located near the Fermi level below zero energy, i bands to the f bands at both wavelengths. Importantly there are no resonance transitions around the *Γ* point at the wavelengths considered.

We conclude from Fig. 3(b) of ref. 42, that there is only one resonant transition for each photon energy (*E* = 1.138 eV and *E* = 1.165 eV) located in the middle of the path *Γ* → *K* in the same segment of the Brillouin zone. Although, in the two transitions considered the DOS in the initial bands i are comparable, the DOS of the f band, corresponding to the resonance transition at *E* = 1.138 eV is much smaller than that of the DOS to f band corresponding to the resonance with transition energy *E* = 1.165 eV. Indeed, whereas the f band for the first transition has a sharp slope, in the second transition the f band is nearly flat. We consider these two transitions and apply the approach above, developed to calculate the dependence of transmittance *T* on the excitation intensity.

In the calculations, we have used for the parameters employed in the problem, values close to those of the experiments on nonlinear absorption. The values for *τ*_i_ and *τ*_f_ for the pump wavelength *λ* = 1000 nm were taken from ref. 14 and 16. We remark that there are some discrepancies between the measured values of the relaxation times in MXenes. However, as our simulations show, the nonlinear absorption characteristics are not very sensitive to variations of the time constants, even if their values are doubled, or halved. For *T*_2_ we have adopted the value of the electron–electron intraband scattering time, as it lies very close to the dipole dephasing time (see ref. 46 and 48 for semiconductors and ref. 50 for metals). The parameter *γ* for the wavelength specified and the reduced effective mass *µ* are determined from the band structure of MXene for thick samples.^14^ The values of parameters used in our calculations are presented in Table 1.

**Table 1:** Values of *γ* and *µ* for *λ* = 1090 nm and *λ* = 1064 nm were determined using DFT calculations from ref. 42. The relaxation times were taken from ref. 14 and 16

Ref.	*λ* [nm]	Δ*T*_pulse_	*γ*	*τ*_e–e,i_ = *τ*_e–e,f_	*τ*_e–ph,i_ = *τ*_e–ph,f_	*µ*	*z*_0_
38	1090	2 µs	0.105	1.0 ps	20 ps	0.101*m*_e_	70–80 nm
39	1064	20 ns	0.998	1.0 ps	20 ps	0.283*m*_e_	14.1 µm

**Fig. 2:**
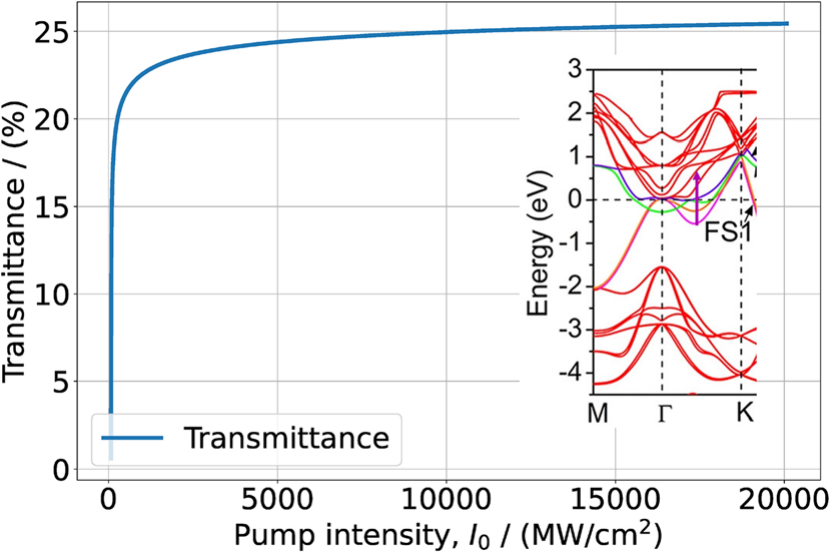
Transmittance of a 70–80 nm thick Ti_3_C_2_T_*x*_–PVA film as function of the excitation intensity at *λ* = 1090 nm. In the inset, the cyan arrow indicates the resonance transition and superposes a fraction of the band structure extracted from Fig. 3(b) of ref. 42. Reprinted and adapted with permission, Copyright (2015) Springer Nature Publishing Group.

**Fig. 3:**
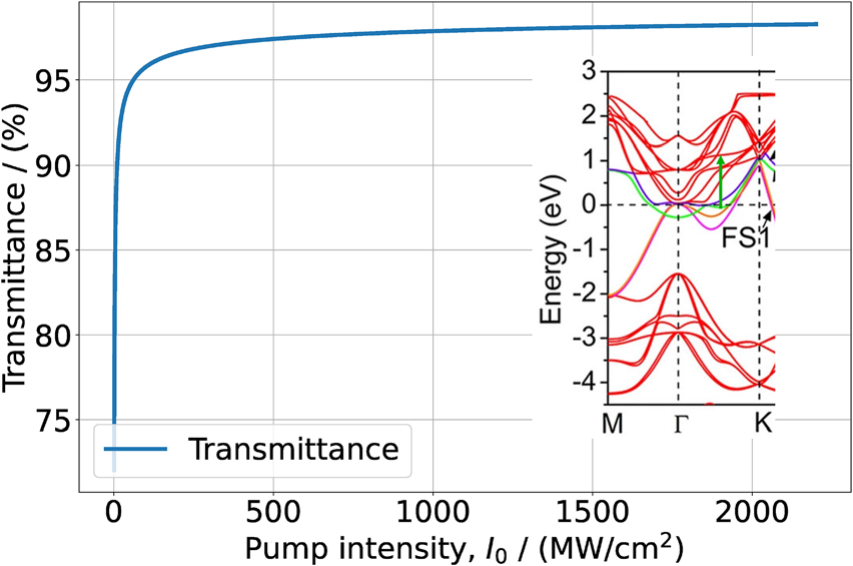
Transmission of Ti_3_C_2_T_*x*_–PVA film as function of the excitation intensity at *λ* = 1064 nm. The green arrow in the inset indicates the resonance transition and superposes a fraction of the band structure extracted from Fig. 3(b) of ref. 42. Reprinted and adapted with permission, Copyright (2015) Springer Nature Publishing Group.

The value *d*_0_ = 1.64 × 10^−27^ C m was calculated from eqn (6) using the experimental data of *α*_0_(*ω*). Our calculations show that the NLA coefficient is insensitive to the choice of *T*_2_, at least for *T*_2_ > 0.5 ps. Therefore, small differences between the results of various studies regarding the measurements of relaxation times cannot significantly affect the results of our calculations. The solutions of eqn (8) for the wavelengths *λ* = 1090 nm and *λ* = 1064 nm allowed to calculate the absorbances and obtain the dependences *T*(*I*_0_), which are presented in Fig. 2 and 3.

We see that while at *λ* = 1090 nm the saturation effect becomes pronounced at pump intensities ≥5000 MW cm^−2^, at *λ* = 1064 nm it manifests itself already at a few MW cm^−2^ in good agreement with experimental data. The comparison between the curves of Fig. 2 and 3 (obtained without the fitting procedure), with the corresponding ones from the experimental publications, show good agreement, with a small deviation of a few percent, with the saturated transmittances measured. Thus, in thick Ti_3_C_2_T_*x*_ samples the saturation regime at *λ* ∼ 1100 nm pump wavelength is fully governed by the DOS of resonant interband transitions. Although the two wavelengths are rather close, nevertheless large differences of DOS in the f bands cause large differences in the saturation regimes. We underline that we do not account for lattice defects and terminations in the saturation intensity, non-saturable losses, usually occurring due to non-resonant absorption processes.

### 3.2. Analysis of the experimental NLA data at pump wavelengths 1550 nm and 1565 nm

To interpret the experimental data of ref. 35 for a Ti_3_C_2_T_*x*_–PVA film, composed of few layer MXene structures for pump wavelengths *λ* = 1565 nm (0.792 eV), we involve the band structures of bilayered Ti_3_C_2_T_*x*_ (Fig. 1). In the case of the multilayered structure considered in ref. 37 at pump wavelength *λ* = 1550 nm (0.8 eV), we exploit the band structure of a stacked MXene (Fig. 3(b) in ref. 42).

**Fig. 4:**
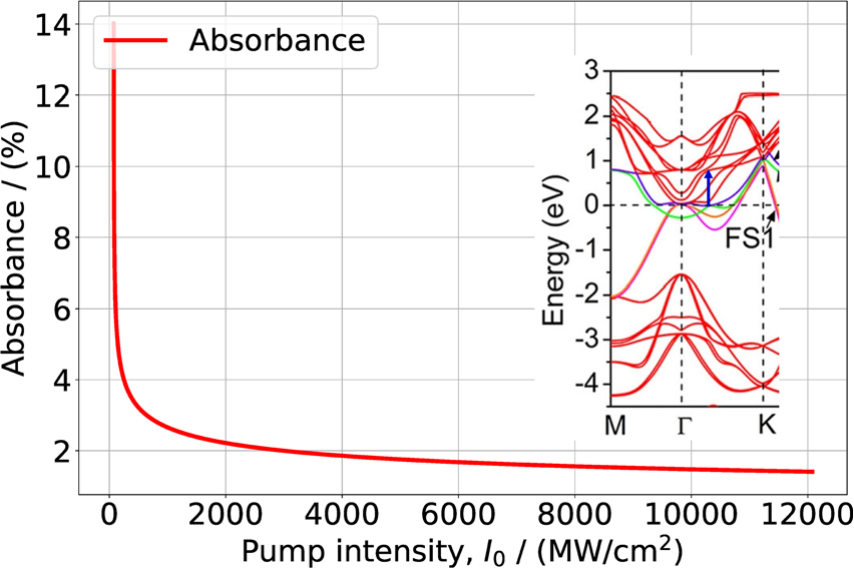
Absorbance of Ti_3_C_2_T_*x*_ in a PVA film as function of the excitation intensity at *λ* = 1550 nm. In the inset the blue arrow indicates the resonance transition, overlapped with a fraction of the band structure of Fig. 3(b) in ref. 42. Reprinted and adapted with permission, Copyright (2015) Springer Nature Publishing Group.

**Fig. 5:**
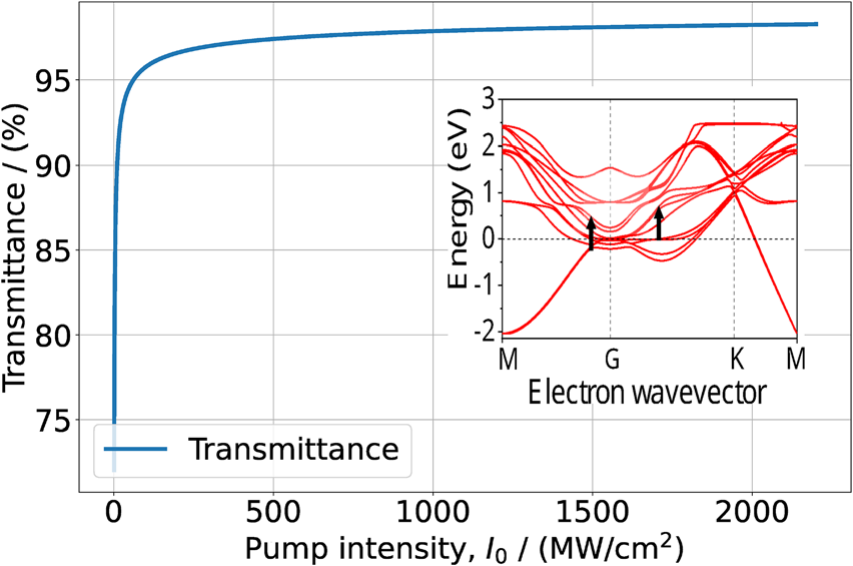
Transmission of film composed of a few layer Ti_3_C_2_T_*x*_ and PVA depending on input intensity at *λ* = 1565 nm. In the inset the black arrows indicate the resonance transition, overlapped with the upper part of the band structure of Fig. S2(c) in ref. 42. Reprinted and adapted with permission, Copyright (2015) Springer Nature Publishing Group.

Furthermore, in Fig. 1 we can see that around the *Γ* point there are no resonance transitions at *λ* = 1550 nm. Instead, at that wavelength there is a resonance transition with high DOS in the f band in the middle of the path *Γ* → *K* of the Brillouin zone. Interestingly, at *λ* = 1565 nm pump wavelength in the band structure of the bilayered sample (Fig. 1) there are two resonance transitions: one around the *Γ* point and the other in the middle of the path *Γ* → *K*. However, the corresponding DOS of the f bands for both transitions is relatively small. Thus, for the pump wavelength at *λ* = 1550 nm we solved eqn (9) with just one resonance transition, whereas for *λ* = 1565 nm we solve eqn (12) with two resonance transitions. The values of *τ*_i_ and *τ*_f_ for the pump wavelength *λ* = 1500 nm were taken from ref. 16. As in the previous case for the quantity *T*_2_ we adopt the time of electron–electron intraband scattering value. The values of other parameters used in the calculations are presented in Table 2. The results of the solution of eqn (8) and eqn (11) for the dependence of transmittance, or absorbance on the input intensity, are presented in the Fig. 4 and 5.

**Table 2:** Values of *γ* and *µ* for *λ* = 1550 nm and *λ* = 1565 nm were determined from DFT calculations.^42^ The relaxation times were taken from ref. 14 and 16

Ref.	*λ* [nm]	Δ*T*_pulse_	*γ*	*τ*_e–e,i_ = *τ*_e–e,f_	*τ*_e−ph,i_ = *τ*_e−ph,f_	*µ*	*z*_0_
35	1550	3 µs	0.092	0.62 ps	33 ps	0.511*m*_e_	Film of Ti_3_C_2_T_*x*_ in PVA
37	1565	2.1 ps	0.351	0.62 ps	33 ps	0.102*m*_e_	Few layers of Ti_3_C_2_T_*x*_ in PVA

It is easy to see that the calculated dependences again agree well with the measured data. Similarly to the case of pump wavelength *λ* ∼ 1000 nm for wavelength *λ* ∼ 1500 nm the saturation behavior strongly depends on the band structure. For pump wavelengths with a separation within 15 nm around *λ* = 1550 nm one would expect the same saturation regime. However, as DFT calculations show, around *λ* ∼ 1550 nm the dispersion properties of electrons are somewhat different in a few-layered and in multilayer samples (see Fig. 3(b) in ref. 42). Namely, for a thick sample, resonant transitions at the indicated wavelength have much larger DOS. This explains why around *λ* ∼ 1550 nm the saturation regime strongly depends on the thickness of the sample.

## 4. Conclusions

We have developed a theoretical model of NLA in the vis-NIR spectral range for titanium carbide MXene employing the band structure. The calculations were carried out using density matrix theory for a two band model in the resonance approximation. Our approach avoids fitting procedures for the determination of nonlinear characteristics of the material and interprets recent experimental results with high accuracy. The latest experimental results on nonlinear absorbance and transmittance at pump wavelengths *λ* ∼ 1000 nm and *λ* ∼ 1500 nm are interpreted in the frame of a microscopic approach, and the saturation dynamics of absorption are revealed. It was found experimentally that two drastically different saturation regimes of NLA for very closely located pump wavelengths, arise. This behavior is interpreted in terms of features of the band structure of the Ti_3_C_2_T_*x*_ MXene. Particularly, for very close laser wavelengths, resonant coupling takes place between different pairs of i and f bands with noticeably different DOS. The method developed can be applied for the investigation of nonlinear characteristics of other MXenes, that will allow identification of new interband resonances with high DOS and realize mode-locking for femtosecond pulse generation at other wavelengths as well.

## Author contributions

The manuscript was written through contributions of all authors. All authors have given approval to the final version of the manuscript.

## Conflicts of interest

The authors declare no competing financial interest.

## Data Availability

The authors declare that the data supporting the findings is available within the paper. The data reproduced in Fig. 1 was provided by the authors of ref. 42.
